# Natalizumab treatment for multiple sclerosis: Middle East and North Africa regional recommendations for patient selection and monitoring

**DOI:** 10.1186/1471-2377-14-27

**Published:** 2014-02-12

**Authors:** Raed A Alroughani, Hany M Aref, Saeed A Bohlega, Maurice P Dahdaleh, Imed Feki, Mohammed A Al Jumah, Muhammad Z Al-Kawi, Salam F Koussa, Mohamad A Sahraian, Isa A Alsharoqi, Bassem I Yamout

**Affiliations:** 1 Amiri Hospital, Arabian Gulf Street, 73767 Kuwait City, Kuwait; 2 Dasman Diabetes Institute, Al-Soor Street, Kuwait City, Kuwait; 3 Ain Shams University, Cairo, Egypt; 4 King Faisal Specialist Hospital and Research Centre, Riyadh, Saudi Arabia; 5 Al Khalidi Hospital, Amman, Jordan; 6 Habib Bourguiba University Hospital, Sfax, Tunisia; 7 KAIMRC, king Saud Ben Abdulaziz university for Health sciences, NGHA and MS center, prince Mohammed Ben Abdulaziz hospital, MOH, Riyadh, Saudi Arabia; 8 Hôtel-Dieu de France Hospital, St Joseph University, Beirut, Lebanon; 9 MS Research Center, Neuroscience Institute, Tehran University of Medical Sciences, Tehran, Iran; 10 Ibn Al-Nafees Hospital, Manama, Bahrain; 11 MS Center, American University of Beirut Medical Center, Beirut, Lebanon

**Keywords:** Multiple sclerosis, Natalizumab, Progressive multifocal leukoencephalopathy, Recommendations, Risk stratification

## Abstract

**Background:**

Natalizumab, a highly specific α4-integrin antagonist, , has recently been registered across the Middle East and North Africa region. It improves clinical and magnetic resonance imaging (MRI) outcomes and reduces the rate of relapse and disability progression in relapsing-remitting multiple sclerosis (MS). Natalizumab is recommended for patients who fail first-line disease-modifying therapy or who have very active disease. Progressive multifocal leukoencephalopathy is a rare, serious adverse event associated with natalizumab.

We aim to develop regional recommendations for the selection and monitoring of MS patients to be treated with natalizumab in order to guide local neurological societies.

**Methods:**

After a review of available literature, a group of neurologists with expertise in the management of MS met to discuss the evidence and develop regional recommendations to guide appropriate use of natalizumab in the region.

**Results:**

Disease breakthrough is defined as either clinical (relapse or disability progression) or radiological activity (new T2 lesion or gadolinium-enhancing lesions on MRI), or a combination of both. Natalizumab is recommended as an escalation therapy in patients with breakthrough disease based on its established efficacy in Phase III studies. Several factors including prior immunosuppressant therapy, anti-John Cunningham virus (JCV) antibody status and patient choice will affect the selection of natalizumab. In highly active MS, natalizumab is considered as a first-line therapy for naive patients with disabling relapses in association with MRI activity. The anti-JCV antibody test is used to assess anti-JCV antibody status and identify the risk of PML. While seronegative patients should continue treatment with natalizumab, anti-JCV antibody testing every 6 months and annual MRI scans are recommended as part of patient monitoring. In seropositive patients, the expected benefits of natalizumab treatment have to be weighed against the risks of PML. Clinical vigilance and follow-up MRI scans remain the cornerstone of monitoring. After 2 years of natalizumab therapy, monitoring should include more frequent MRI scans (every 3–4 months) for seropositive patients, and the risk-benefit ratio should be reassessed and discussed with patients.

**Conclusions:**

Recommendations have been developed to guide neurologists in the Middle East and North Africa on patient selection for natalizumab treatment and monitoring.

## Background

Multiple sclerosis (MS) is an inflammatory demyelinating disorder affecting the central nervous system. Historically, the Kurtzke classification has designated many Middle Eastern countries as being low-risk zones for MS, but recent studies have shown that the crude incidence of MS in this region ranges from 31–85 per 100,000 individuals, and that the annual incidence is increasing [1-4]. Interferon-beta is generally regarded as the first-line treatment for MS, with natalizumab and fingolimod used as second-line agents in the case of treatment failure with interferon-beta [1].

Natalizumab, a highly specific α4-integrin antagonist, has recently been registered across the Middle East and North Africa (MENA) region. It is recommended as a single disease-modifying therapy (DMT) in patients with highly active relapsing-remitting MS to prevent relapses and delay progression of disability. Natalizumab is indicated in patients who fail to respond to a full and adequate course (normally at least 1 year of treatment) of interferon-beta or glatiramer acetate, or for those who have rapidly evolving, severe relapsing-remitting MS. Failure to respond is defined as at least one relapse in the previous year on therapy and at least nine T2-lesions in cranial magnetic resonance imaging (MRI) or at least one gadolinium-enhancing (Gd) lesion. Rapidly evolving severe relapsing-remitting MS can be defined by at least two disabling relapses in 1 year and at least one Gd-enhancing lesion on brain MRI or a significant increase of T2 burden [5].

Natalizumab improves clinical and MRI outcomes and reduces the rate of relapse and disability progression in relapsing-remitting MS [6-9]. The Phase III AFFIRM (Natalizumab Safety and Efficacy in Relapsing-Remitting MS) study showed that monotherapy with natalizumab for 2 years decreased the annualised relapse rate (ARR) by 68% (p < 0.001) and the disability progression rate (sustained for 3 months) by 42% (p < 0.001) compared with placebo [6]. For MRI outcomes, a 92% decline in the number of Gd-enhancing lesions during the second year (p < 0.001), an 83% decrease in the number of new or enlarging T2-hyperintense lesions over 2 years (p < 0.001), and a 76% fall in new T1-hypointense lesions were seen [7]. Natalizumab was found to be generally safe and well tolerated [6]. The advent of newer therapies such as natalizumab has led to more ambitious aims of MS treatment, with freedom from measured disease activity and improvement being possible [10,11].

Progressive multifocal leukoencephalopathy (PML) is a rare, serious adverse event associated with natalizumab. PML is an opportunistic infection of the central nervous system that is caused by John Cunningham virus (JCV). The virus replicates in glial cells of the brain, leading to lytic death of oligodendrocytes. PML is very rare in immunocompetent people, typically occurring in severely immunocompromised patients such as those with HIV infection, malignant disease or organ transplants. It may present with any combination of weakness, speech disturbances, limb incoordination, cognitive deficits and visual impairment, usually resulting in death or severe disability [12]. Three cases of PML were identified in the pivotal natalizumab clinical trials.

Post-marketing reports of PML cases have been systematically collected, and risk management strategies have been developed on the basis of an increased risk in patients with seropositive anti-JCV antibodies, with longer duration of natalizumab treatment and with prior immunosuppressive therapy [13]. The risk of PML among patients with none of these risk factors is very low. There is now evidence to suggest that outcomes of PML may be better in patients in whom the condition is detected and treated early, even before the presence of clinical symptoms [14].

Our aim is to guide local neurological societies in the MENA region by developing recommendations for the selection and monitoring of MS patients to be treated with natalizumab.

## Methods

After a review of available literature, a group of neurologists with expertise in the diagnosis and management of MS met to discuss the updated evidence and to develop regional recommendations to guide appropriate use of natalizumab in the region. The panel used the revised 2010 Mcdonald diagnostic criteria for MS in the discussion and recommendations [15]. The neurologists reviewed evidence on the efficacy of natalizumab in comparison with other MS therapies, and on the risk of PML and its early detection and management. Relevant cases were discussed to highlight clinical decision points, and agreement was reached on key topics through voting on a series of questions on the positioning of natalizumab in the MS treatment paradigm. Several important factors were discussed, including appropriate patient selection, routine safety monitoring, and an understanding of both early recognition and timely management of PML. Disease breakthrough was defined as either clinical (relapse or disability progression) or radiological activity (new T2 lesion or Gd-enhancing lesions on MRI) or a combination of both. Relapse was defined as new or recurrent neurologic symptoms not associated with fever or infection that lasted for at least 24 hours and were accompanied by new neurological signs found by the examining neurologist. Disease progression was defined as an increase of at least 1.5 points on the Expanded Disability Status Scale (EDSS) score [16]. Disabling relapse was defined as a relapse with a residual disability, which was defined as incomplete recovery at 1 month from a relapse [17,18]. As the panel did not conduct any novel experimental research, no ethics approval was necessary.

## Results and discussion

### Efficacy of natalizumab

The goals of MS treatment have evolved as new therapies have become available. While initial therapies reduced symptoms and the next generation slowed disease progression, the current aim is to stop disease progression and to achieve freedom from disease activity [10]. Sustained improvement in neurological disability has been shown to be possible [11]. An EDSS score of 3 seems to be a turning point in the development of disability, with progression being more rapid and difficult to stop after that point [19]. Key decision points in MS treatment are therefore initiation of therapy and escalation of therapy.

While results from different clinical studies cannot be directly compared, the 68% reduction in ARR seen with natalizumab [6] is higher than those reported for glatiramer acetate and interferon-beta 1a and 1b (29–34%) [20-23] or fingolimod (54%) [24]. Similarly, natalizumab reduced disability progression by 42% sustained at 12 weeks and by 54% sustained at 24 weeks [6], whereas fingolimod performed comparably to interferon (37% reduction sustained at 24 weeks with both) [23,24]. In a subgroup analysis of AFFIRM data, patients with very active MS (>2 relapses in previous year and Gd-enhancing lesions at baseline) had reductions of 81% in ARR, and 53% and 64% reduction in sustained (12 and 24 weeks, respectively) disability progression with natalizumab [25]. Equivalent figures for fingolimod were 63% and 22% [26]. Five times more patients were free from disease activity with natalizumab versus placebo (37% vs. 7%) compared with 2.5 times for fingolimod (33% vs. 13%) [10].

The efficacy of natalizumab reported from the clinical trials is supported by real-world data. The Tysabri Observational Program (TOP) is an open-label, observational study being conducted in Europe, Australia and Canada, with 3976 patients from 15 countries enrolled by December 2011. The ARR was reduced from 1.99 (95% CI 1.96–2.03) at baseline to 0.26 (95% CI 0.23–0.30; p < 0.0001) after 3 months of natalizumab treatment and was maintained at that rate at 4 years [27]. EDSS was stable over 4 years, in both patients with a high (4.5) or low (2.0) starting median EDSS, and more patients had confirmed improvement rather than disability progression [28]. In the TYNERGY study, natalizumab was prescribed in a real-life setting, and the Fatigue Scale for Motor and Cognitive Functions (FSMC) showed a reduction in 9.0 points (p < 0.0001) after 12 months, corresponding to a reduction from severe to moderate fatigue [29]. Scores for quality of life, sleepiness, depression, cognition and disability progression were all improved from baseline (all p < 0.0001), and walking speed as measured by the 6-minute walk-test also increased at month 12 (p = 0.0016) [29]. In the Swedish IMSE (Immunomodulation and Multiple Sclerosis Epidemiology) study, a significant improvement in Symbol Digit Modalities Test (SDMT) score (from 49.7 to 55.6; p < 0.05) was seen over 24 months’ natalizumab treatment [30]. In a Belgian cohort, natalizumab increased freedom from disease activity in relapsing-remitting MS patients after previous therapy failure, with 62% being free of disease activity after 44 weeks. EDSS was marginally worse in 4% of patients, stable in 40%, marginally improved in 27% and improved by ≥1 point in 29% [31].

There is no level 1 evidence to support the use of natalizumab in patients under the age of 18 years, and this therapy is not licensed for use in paediatric patients. However, some studies have evaluated its use in paediatric cohorts [32-34]. In an Italian cohort of 55 patients (mean age 14.4 years), significant improvement in mean EDSS scores was reported (from 2.7 to 1.9 at the last visit; p < 0.001) [32]. Similarly, in a Spanish cohort of nine patients (mean age 15.3 years), eight demonstrated a median improvement in EDSS score from 3.0 to 1.0, and a reduction in median ARR from 3.0 to 0 [33]. Natalizumab also appears to be generally well tolerated in this patient population [34]. In light of this evidence, the experts suggest that the use of natalizumab would need to be carefully considered and discussed on a case-by-case basis.

### Discontinuation of natalizumab

When considering the withdrawal of natalizumab therapy, the physician should consult with patients on an individual basis, reassess the benefit/risk of natalizumab versus burden of disease and alternative therapies, and increase alertness and monitoring. Studies evaluating the effect of natalizumab interruption show that MS activity may return to pre-treatment levels within 4–7 months, and that the levels of disease activity did not exceed those noted during the trials in subjects randomised to placebo [35]. The pattern of the return of MS disease activity was consistent with known pharmacokinetic and pharmacodynamic properties of natalizumab and did not show evidence of rebound [35].

### PML risk assessment

**Figure 1:**
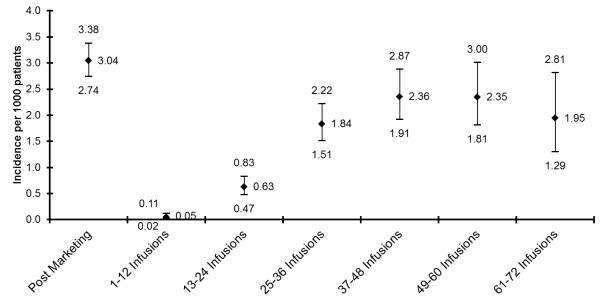
**Natalizumab PML risk estimates by treatment epoch**[37]**.** The observed clinical trial PML incidence in patients who received a mean of 17.9 monthly doses of natalizumab was 1.00 per 1000 natalizumab-treated patients (95% CI 0.20–2.80) [36]. The post-marketing rate is calculated as the number of PML cases in patients who have had at least one dose of natalizumab. Incidence estimates by treatment epoch are calculated based on natalizumab exposure through 30 April 2013 and 359 confirmed cases as of 6 May 2013. The incidence for each epoch is calculated as the number of PML cases divided by the number of patients exposed to natalizumab.

The observed PML incidence in patients who received a mean of 17.9 monthly doses of natalizumab in the clinical trials was 1.00 per 1000 natalizumab-treated patients (95% CI 0.20–2.80) [36]. The post-marketing rate is calculated as the number of PML cases in patients who have had at least one dose of natalizumab. Worldwide post-marketing data from 23 November 2004 to 31 March 2013 comprised 279,196 patient-years of natalizumab exposure. Overall, 115,400 patients were treated in this period: 83,000 patients for at least 12 months, 59,100 for at least 24 months, 41,100 for at least 36 months and 26,600 for at least 48 months. As of 6 May 2013, 359 confirmed cases of PML had occurred, giving an overall post-marketing rate of 3.04 (95% CI 2.74–3.38) per 1000. The rate increases with number of natalizumab infusions, from 0.05/1000 with 1–12 infusions to >2/1000 after 36 infusions, with a tripling of risk at around 24 infusions (Figure 1). Of the 359 cases, 123 were from the US, 212 from Europe and 24 from the rest of the world; 83 (23%) patients died and 276 (77%) were still alive as of 6 May 2013 [37].

JCV infection is one of the key factors for PML development. A two-step enzyme-linked immunosorbant assay (ELISA) has been developed to detect the presence of anti-JCV antibodies in serum or plasma to help identify patients who have been exposed to JCV [38]. This assay is now being used as a risk stratification tool in natalizumab-treated patients. The prevalence of anti-JCV antibodies in MS patients is approximately 50–60% [38]. Preliminary data suggest that 2–3% of patients seroconvert annually (i.e. change from anti-JCV antibody negative status to positive status and remain positive over time) [39]. Of the confirmed PML cases, 139 had pre-PML samples available for anti-JCV antibody testing and 138 of these tested positive prior to diagnosis of PML (6–187 months). One patient tested negative 9 months before PML diagnosis, and no further samples were available; another patient tested negative 9 months before PML diagnosis and positive 6.5 months before diagnosis [37].

**Figure 2:**
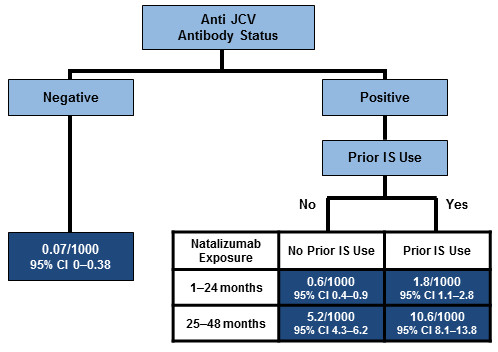
**PML risk stratification in natalizumab-treated MS patients**[37]**.** Based on natalizumab exposure and 285 confirmed PML cases as of 5 September 2012. Prior immunosuppression (IS) data in overall natalizumab-treated patients based on proportion of patients with IS use prior to natalizumab therapy in TYGRIS as of May 2011; and prior IS data in PML patients as of 5 September 2012. The analysis assumes that 55% of natalizumab-treated MS patients were anti-JCV antibody positive and that all PML patients test positive for anti-JCV antibodies prior to the onset and diagnosis of PML. The estimate of PML incidence in anti-JCV antibody negative patients is based on the assumption that all patients received at least 1 dose of natalizumab. Assuming that all patients received at least 18 doses of natalizumab, the estimate of PML incidence in anti-JCV antibody negative patients was generally consistent (0.1/1000; 95% CI 0.00–0.62).

Risk stratification based on natalizumab exposure, anti-JCV antibody status and prior immunosuppressive therapy offers an opportunity for personalised management of MS patients. Figure 2 shows the stratification of PML risk in patients treated with natalizumab. It can be seen that the risk of PML in anti-JCV antibody negative patients is very low (0.07/1000); duration of therapy and immunosuppressive history are not relevant factors in this patient segment.

### Benefits of early PML detection and treatment

Neurologists need to exercise clinical vigilance for the signs and symptoms of PML. Onset is usually subacute, occurring over several weeks, and is progressive (compared with the typical acute onset of an MS relapse). Clinical presentation can include visual change and motor or behavioural changes [14]. Recent changes in behaviour or personality, hemiparesis, language disturbances, retrochiasmal visual deficits and onset of seizures should alert the clinician to possible PML. Relatives may notice subtle changes that are not apparent to the clinician. PML lesions can be detected on MRI scans. In a review of 22 of the first 40 post-marketing cases of PML, the most frequent lesion pattern in early scans from PML patients was that of large (>3 cm, 15/18), subcortical (18/18), T2 or fluid-attenuated inversion recovery hyperintense (18/18), T1-hypointense (17/18), and diffusion-hyperintense (15/15) lesions, with a sharp border towards the grey matter and an ill-defined border toward the white matter (18/18) on T2-weighted images. Contrast enhancement was detected in 41% (7/17) of the cases on the first scan at clinical presentation [40]. A diagnosis of PML can usually be confirmed by cerebrospinal fluid (CSF) analysis using JCV DNA amplification by the polymerase chain reaction (PCR) technique with a sensitivity of 80% and a specificity approximating 100% [12].

PML is treated by immune reconstitution. Plasma exchange rapidly reduces natalizumab concentrations in serum and restores leukocyte function [41]. Several courses of plasma exchange or immunoadsorption have been used to eliminate natalizumab in post-marketing PML cases [42]. However, this treatment is often followed by immune reconstitution inflammatory syndrome (IRIS), which can lead to serious neurological complications or death. IRIS can be successfully treated with high-dose steroids [13]; prophylaxis of IRIS with corticosteroids has not been systematically evaluated, but given the severe nature of IRIS and its consistent presentation in most patients, pre-emptive treatment starting immediately after plasma exchange might be justified [43].

Factors that appear to be associated with improved survival in PML patients include younger age at diagnosis, lower pre-PML EDSS score, shorter time from first symptoms to diagnosis and localised PML extension on MRI at diagnosis (i.e. unilobar vs. multilobar or widespread lesions) [44]. Recently, several cases have been reported in which PML has been detected by MRI in asymptomatic patients [45,46]. In a study of patients with natalizumab-associated PML, 7% (21/298) were asymptomatic at the time of diagnosis [14]. Most of these (14/21) remained symptom-free over 13 months of follow-up. Asymptomatic patients experienced significantly less functional disability at diagnosis and at 6 and 12 months post-diagnosis compared with symptomatic PML patients. Over time, EDSS scores were consistently better in asymptomatic PML patients than in symptomatic patients. As of 1 January 2013, 100% of asymptomatic PML patients and 77% of symptomatic PML patients were alive [14]. These preliminary data suggest that functional outcomes and survival may be better in patients whose PML is diagnosed before they develop clinical symptoms.

### Clinical management and positioning of natalizumab

The expert neurologists considered descriptions of several clinical cases and discussed their management. They then voted on a series of questions on the positioning of natalizumab in the paradigm of MS therapies. Table 1 summarises the conclusions on the positioning of natalizumab relative to other MS therapies.

**Table 1:** Summary of positioning of natalizumab in the paradigm of MS therapies

**Indication**	**Clinically isolated syndrome**	**Relapsing-remitting MS**	
Escalation therapy		Natalizumab	
		Fingolimod (depending on other risk factors: anti-JCV antibody status, diabetes mellitus, cardiac problems)	
		Failure to other treatments (e.g. mitoxantrone, cyclophosphamide)	
Baseline therapy	Glatiramer acetate	Glatiramer acetate	*Highly active patients*:
	Interferon	Interferon	Natalizumab
			Fingolimod

### Regional recommendations

**Figure 3:**
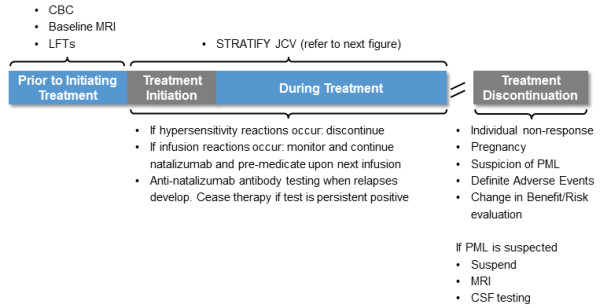
**Regional recommendations for maximising safety of natalizumab.** CBC: complete blood count; CSF: cerebrospinal fluid; JCV: John Cunningham virus; LFTs: liver function tests; PML: progressive multifocal leukoencephalopathy.

**Figure 4:**
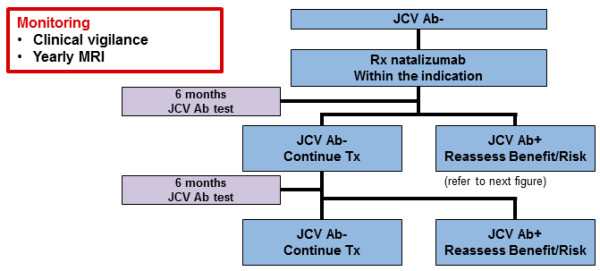
**Regional recommendations for using natalizumab in anti-JCV antibody-negative patients.** JCV: John Cunningham virus.

**Figure 5:**
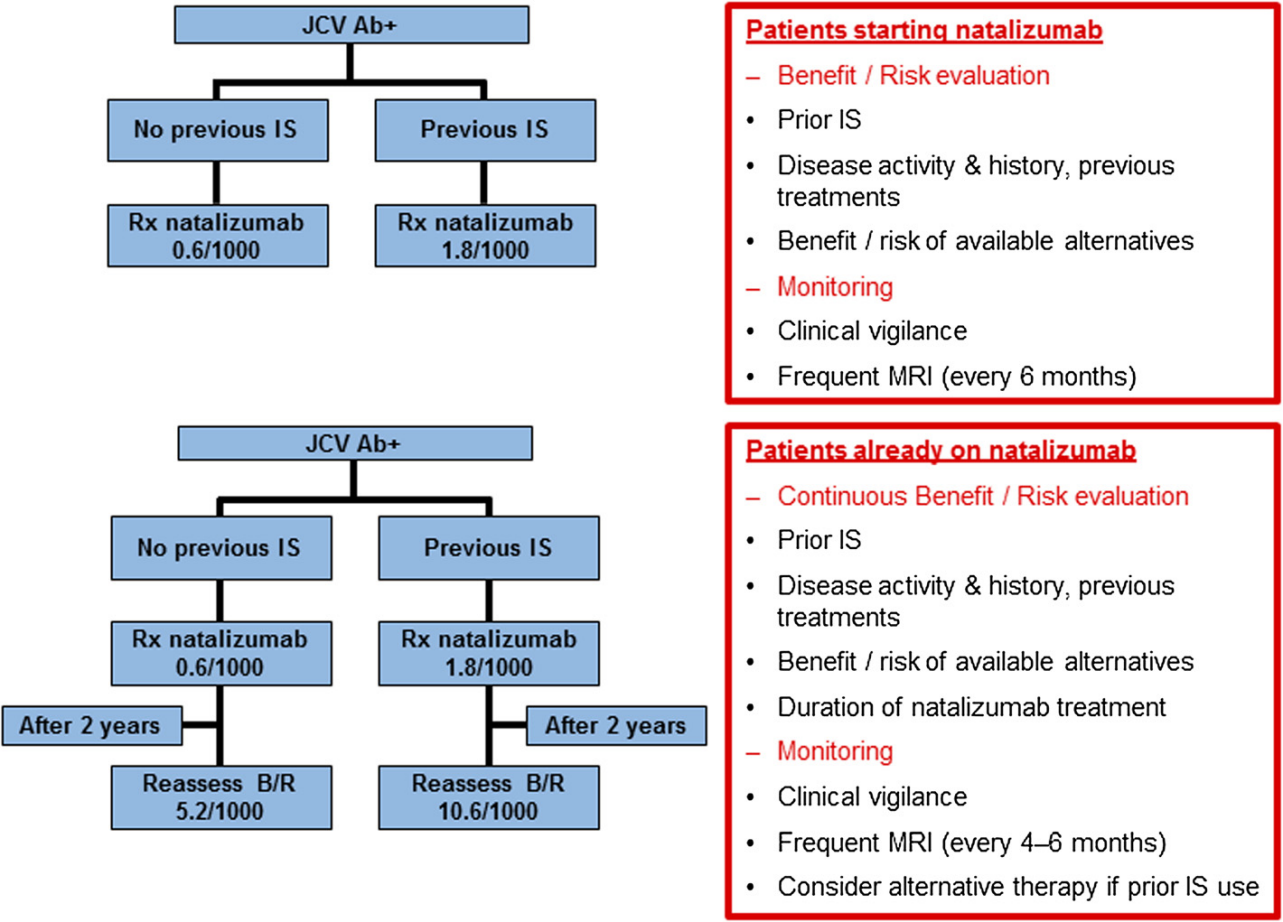
**Regional recommendations for using natalizumab in anti-JCV antibody-positive patients.** B/R: Benefit/risk; IS: immunosuppression; JCV: John Cunningham virus.

Figures 3, 4 and 5 show the algorithm developed during the discussion. Natalizumab is recommended as an escalation therapy in patients with breakthrough disease on the basis of its established efficacy in Phase III studies. In highly active MS, natalizumab is considered as a first-line therapy for naive patients with disabling relapses in association with MRI activity. Several factors including prior immunosuppressant therapy, anti-JCV antibody status and patient choice may contribute to the selection of natalizumab. A complete blood count and a cranial MRI scan (within 3 months of starting natalizumab therapy) are recommended at baseline. This enables comparison with subsequent scans that may be done to investigate the cause of new or worsening neurological symptoms once on natalizumab therapy. Figure 3 shows recommendations for maximising the safety of natalizumab. Treatment should be stopped if hypersensitivity reactions or persistent anti-drug antibodies occur. If infusion reactions occur, treatment can be continued with monitoring and pre-medication. Treatment is discontinued in the event of non-response, pregnancy, suspicion of PML, definite adverse events or a change in the benefit–risk evaluation. A low threshold to withhold natalizumab and investigate with MRI and CSF testing is recommended when a diagnosis of possible PML is entertained. If a thorough neurological assessment cannot rule out PML, natalizumab must be suspended and not restarted until a disorder other than MS has been excluded with confidence. Natalizumab can be resumed only if the diagnosis of PML is discounted [43].

The anti-JCV antibody test is used to assess anti-JCV antibody status. It is recommended to obtain the test at treatment initiation or during treatment with natalizumab, although the results will be unlikely to alter the management decision in the first 12–24 months. Anti-JCV antibody testing every 6 months and annual MRI scans are recommended during natalizumab treatment (Figure 4). Seronegative patients should continue treatment with natalizumab. In seropositive patients, the expected benefits of natalizumab treatment have to be weighed against the risks. Clinical vigilance and follow-up MRI scans are recommended as monitoring parameters (Figure 5). After 2 years of natalizumab therapy, monitoring should include more frequent MRI scans (every 3–4 months), and the benefit–risk ratio of natalizumab treatment should be reassessed and discussed with patients. The increased risk of PML with previous immunosuppressant use seems to be independent of PML risk associated with duration of natalizumab treatment. Risk stratification by previous immunosuppressant use and treatment duration must be considered in a broader context that includes additional factors such as benefits of treatment, risks of inadequately treated MS or progression of the disease, and the relative benefit–risk profiles of alternative treatments [13]. Anti-JCV antibody status seems to be stable over time, with an annual seroconversion rate of approximately 2–3% [39]. Specific education of physicians with respect to management of PML is needed for all prescribers.

## Conclusions

These recommendations have been developed by expert neurologists in the MENA region through a process of discussion and voting on key questions. They are intended to guide neurologists in the region in selecting appropriate MS patients for natalizumab treatment and monitoring them to minimise the risk of PML and to detect PML cases early as asymptomatic detection is associated with better outcomes.

## Abbreviations

ARR: Annualised relapse rate; CSF: Cerebrospinal fluid; DMT: Disease-modifying therapy; EDSS: Expanded disability status scale; IRIS: Immune reconstitution inflammatory syndrome; JCV: JC virus; MENA: Middle East and North Africa; MRI: Magnetic resonance imaging; MS: Multiple sclerosis; PML: Progressive multifocal leukoencephalopathy.

## Competing interests

The authors received honoraria from Biologix (the distributor for Biogen Idec across the Middle East and North Africa region) for serving on their advisory board and from other pharmaceutical companies including Novartis, GSK, Bayer and Merck Serono.

## Authors’ contributions

All authors participated as members of the panel of experts in the meeting that led to the development of the manuscript. All authors actively contributed to the discussion and the consensus reached. RA and BY drafted the initial version of the manuscript and all authors discussed and reviewed the final version of the manuscript. All authors read and approved the final manuscript.

## Pre-publication history

The pre-publication history for this paper can be accessed here:

http://www.biomedcentral.com/1471-2377/14/27/prepub
